# Safety of live attenuated influenza vaccine in atopic children with egg allergy

**DOI:** 10.1016/j.jaci.2014.12.1925

**Published:** 2015-08

**Authors:** Paul J. Turner, Jo Southern, Nick J. Andrews, Elizabeth Miller, Michel Erlewyn-Lajeunesse, SNIFFLE Study Investigators, Christine Doyle, George Du Toit, Michel Erlewyn-Lajeunesse, Roisin Fitzsimons, Paul T. Heath, Stephen M. Hughes, Louise Michealis, Jürgen Schwarz, Matthew D. Snape, Gary Stiefel, Huw M. Thomas, Paul J. Turner

**Affiliations:** a Section of Paediatrics (Allergy & Immunology) and MRC & Asthma UK Centre in Allergic Mechanisms of Asthma, Imperial College London, London, United Kingdom; b Immunisation, Hepatitis and Blood Safety Department, Public Health England, London, United Kingdom; c Division of Paediatrics and Child Health, University of Sydney, Sydney, Australia; d Department of Paediatric Allergy & Immunology, University Hospital Southampton NHS Foundation Trust, Southampton, United Kingdom

**Keywords:** Egg allergy, live attenuated influenza vaccine, asthma, recurrent wheezing, safety, BTS, British Thoracic Society, IIV, Inactivated influenza vaccine, IQR, Interquartile range, LAIV, Live attenuated influenza vaccine, SIGN, Scottish Intercollegiate Guidelines Network, UK, United Kingdom

## Abstract

**Background:**

Live attenuated influenza vaccine (LAIV) is an intranasal vaccine recently incorporated into the United Kingdom immunization schedule. However, it contains egg protein and, in the absence of safety data, is contraindicated in patients with egg allergy. Furthermore, North American guidelines recommend against its use in asthmatic children.

**Objective:**

We sought to assess the safety of LAIV in children with egg allergy.

**Methods:**

We performed a prospective, multicenter, open-label, phase IV intervention study involving 11 secondary/tertiary centers in the United Kingdom. Children with egg allergy (defined as a convincing clinical reaction to egg within the past 12 months and/or >95% likelihood of clinical egg allergy as per published criteria) were recruited. LAIV was administered under medical supervision, with observation for 1 hour and telephone follow-up 72 hours later.

**Results:**

Four hundred thirty-three doses were administered to 282 children with egg allergy (median, 4.9 years; range, 2-17 years); 115 (41%) had experienced prior anaphylaxis to egg. A physician's diagnosis of asthma/recurrent wheezing was noted in 67%, and 51% were receiving regular preventer therapy. There were no systemic allergic reactions (upper 95% CI for population, 1.3%). Eight children experienced mild self-limiting symptoms, which might have been due an IgE-mediated allergic reaction. Twenty-six (9.4%; 95% CI for population, 6.2% to 13.4%) children experienced lower respiratory tract symptoms within 72 hours, including 13 with parent-reported wheeze. None of these episodes required medical intervention beyond routine treatment.

**Conclusions:**

In contrast to current recommendations, LAIV appears to be safe for use in children with egg allergy. Furthermore, the vaccine appears to be well tolerated in children with a diagnosis of asthma or recurrent wheeze.

Egg allergy is one of the most common food allergies in childhood, with an estimated prevalence of at least 2% in preschool children.^1^ Influenza vaccines generally contain egg protein (including ovalbumin) because the vaccine virus is cultured in hen's eggs; only vaccines with an ovalbumin concentration of less than 2 μg/mL are currently approved by the United Kingdom (UK) national regulator. In theory, patients with egg allergy might be at increased risk of an allergic reaction to influenza vaccines. In recent years, inactivated influenza vaccines (IIVs) with very low or no ovalbumin content have become available. Observational studies have confirmed the safety of the parenteral IIV in children with egg allergy, including those with a history of previous anaphylaxis to egg,^2,3^ and have led to a relaxation of contraindications relating to egg allergy in some guidelines.^4-6^

A trivalent live attenuated influenza vaccine (LAIV) administered through the intranasal route has been available in the United States for several years and received approval for use in Europe in 2010. The vaccine has high efficacy against influenza in children aged 2 to 17 years,^7,8^ with a similar safety profile to IIV in children without egg allergy.^9-14^ LAIV is also grown in hen's eggs and contains egg proteins. Until recently, there were no published data on the safety of LAIV in children with egg allergy, and thus its use in this population has been contraindicated.

Authorities in North America recommend annual influenza vaccination in children from 2 to 8 years of age, preferably with LAIV.^6^ LAIV is not licensed for use in children less than 2 years of age because of an increased incidence of wheezing in this age group after immunization.^10,15^ This effect has not been seen in older children,^11,15,16^ even in those with pre-existing asthma and wheeze,^9^ a finding confirmed in postmarketing surveillance data.^12,13^ Nonetheless, current guidance from the US Centers for Disease Control and Prevention recommends against using LAIV in children less than 5 years of age with asthma or an episode of wheezing in the previous year.^6^

In 2013, the UK introduced annual influenza immunization using LAIV into the National Immunization Schedule for children.^17^ Given that the rate of egg allergy in this age group is estimated to be 2.5%, we estimate (on the basis of UK 2013 population data) that there are 60,000 children in this age group for whom LAIV is contraindicated because of a diagnosis of egg allergy. Therefore egg allergy is a significant barrier to successful implementation of the immunization program, resulting in a requirement to vaccinate children with egg allergy with IIV administered by means of injection (typically in the hospital environment), something which is less acceptable to families and would incur significantly higher health costs. As a result, we sought to assess the safety of LAIV in children with egg allergy to provide data to inform an evidence-based consideration of a change to current guidelines.

## Methods

We conducted a phase IV open-label study of LAIV in children with egg allergy during the UK influenza season (September 2013 to January 2014) across 12 hospital-based allergy centers in the UK. Study participants were recruited locally from allergy clinics. Eligible participants were aged 2 to 17 years with (1) IgE-mediated food allergy to egg, which was defined as a positive food challenge result to egg within the last 12 months under medical supervision; (2) a previous convincing clinical reaction to egg within the past 12 months with evidence of current sensitization on the basis of a positive skin prick test response or serum-specific IgE level to egg white; or (3) evidence of current sensitization consistent with a greater than 95% likelihood of clinical egg allergy, as per published criteria.^18^ Patients with a history of prior anaphylaxis to egg or a history of severe but stable asthma were not excluded. Anaphylaxis was defined by using World Allergy Organization criteria.^19^ Asthma was classified according to current therapy at the time of immunization using the British Thoracic Society (BTS) and Scottish Intercollegiate Guidelines Network (SIGN) guidelines.^20^ Skin prick testing was performed in all participants before inclusion according to published guidelines to confirm sensitization to egg (egg white extract; ALK-Abelló, Hørsholm, Denmark) and detect sensitization to potential aeroallergens. Testing and vaccination were deferred if participants had received an antihistamine within the previous 4 days. Participants were excluded if they had previously required invasive ventilation for an anaphylactic reaction to egg, had severe unstable asthma, or had a contraindication to LAIV, such as a prior allergic reaction to a vaccine component (other than egg) or current salicylate therapy or had experienced significant immunocompromise. Vaccination was deferred in participants with acute febrile illness or evidence of increased asthma symptoms for at least 2 weeks after symptom resolution.

The study was approved by the West Midlands–Edgbaston Research Ethics Committee (13/WM/0231), and the parent/guardian of each participant provided written informed consent. Children older than 8 years were encouraged to provide their own assent. The study sponsor was the University Hospital Southampton NHS Foundation Trust (study no. RHM CHI0659). This study was registered with ClinicalTrials.gov (NCT01859039) and the European Union Clinical Trials Register (EudraCT 2013-002031-26).

### Procedures

Participants had baseline parameters (blood pressure, heart rate, respiratory rate, and oxygen saturation) measured before LAIV administration, with clinical respiratory and dermatologic assessment at the same time. LAIV (Fluenz [marketed as Flumist in North America] produced for the 2013-2014 influenza season; AstraZeneca, London, UK) was administered into the nasal airway according to the approved summary of product characteristics (ie, 0.1 mL per nostril) in either the allergy day case or clinical research unit at each hospital site. Participants were observed for at least 1 hour for symptoms of local or systemic allergic reactions, as defined by international consensus.^21^ Clinical observations were recorded for 60 minutes after vaccine administration, along with symptom scoring (total ocular and nasal symptom score).^22^ In one center a subset of patients underwent acoustic rhinometry, an objective assessment of nasal airway patency before and 10 minutes after LAIV administration, as previously described.^23^ Emergency contact details were provided for parents to seek advice in the event of any concerns after vaccination. Parents were contacted by telephone after a minimum of 72 hours to detect any delayed adverse reaction.

Participants who had not received immunization with nonpandemic influenza vaccine in previous years were offered a second dose of LAIV at least 4 weeks later in line with the product recommendations.

### Outcomes

The primary outcome was the incidence of allergic reaction as an adverse event after immunization occurring within 2 hours of LAIV administration in children with egg allergy. A systemic allergic reaction (anaphylaxis) was defined according to the Brighton Collaboration case definition.^24^ Secondary outcomes were as follows: incidence of delayed symptoms occurring up to 72 hours after LAIV administration; incidence of adverse events of nonallergic cause after LAIV administration; and change in nasal airway patency in children who underwent acoustic rhinometry as an additional assessment. The causality of all adverse events was confirmed by an independent data monitoring committee in conjunction with the local study team.

### Statistical analyses

Analyses were planned prospectively and detailed in a statistical analysis plan. The incidence of reactions to LAIV (both immediate and delayed) was estimated with 2-sided exact 95% CIs. For subgroup analyses, incidences of reactions were compared between different cohorts by using a 2-sided Fisher exact test. Sample size was considered with respect to a historical comparison and also based on the precision around an estimate of zero. If there were no allergic reactions in a sample size of 300, then this would provide confidence (based on the upper end of the 2-sided 95% CI) that the true rate of allergic reaction to LAIV in children with egg allergy within the population is no more than 1.2%. The analysis data set was as treated and with relevant safety data measured.

## Results

**Fig 1:**
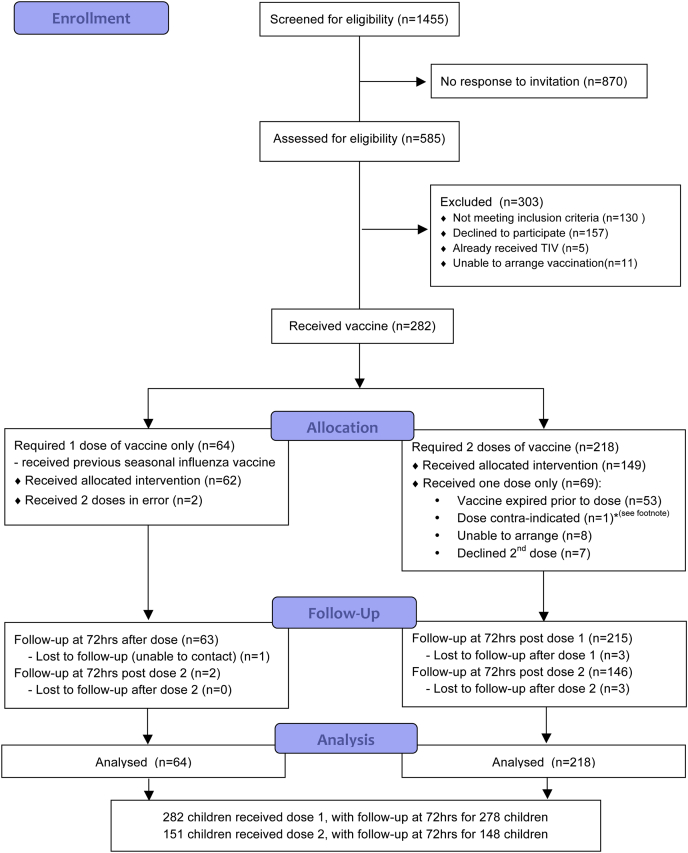
Patient flow diagram. *One child could not receive LAIV because a family member had commenced immunosuppressant therapy for medical reasons. This child was given IIV instead.

Two hundred eighty-two children with egg allergy were enrolled in the study and received at least 1 dose of LAIV between September 2013 and January 2014. The median age of the cohort was 4.9 years (range, 2-17 years; interquartile range [IQR], 3-8 years), and 185 (66%) were male. A total of 433 doses of LAIV were administered to 282 children, 64 with prior influenza vaccination and 218 vaccine-naive children, as depicted in Fig 1. One hundred fifty-one children received a second dose of LAIV 4 weeks later. The reasons for only a single dose of LAIV being administered in the remainder are shown in Fig 1. Unfortunately, 53 children were unable to receive a second dose because of unavailability of in-date vaccine; none of these children were in a high-risk clinical category requiring 2 doses according to UK immunization guidelines.^17^

All children had evidence of current egg allergy at the time of immunization. One hundred forty-five (51%) children experienced an allergic reaction to egg in the last 12 months with evidence of sensitization at enrollment. Twenty-two (8%) had undergone formal, in-hospital food challenges to egg within the previous 12 months to substantiate their diagnosis. A total of 137 (49%) had not reacted to egg in the last 12 months but had evidence of sensitization (ie, greater than the published criteria of >95% positive predictive values for clinical egg allergy).^20^ Only 35 (12%) had never eaten egg and were given a diagnosis based on results of predictive allergy testing alone. The median skin prick test response to egg white was 7 mm (IQR, 5-9 mm; range, 0-16 mm), and the median serum-specific IgE level was 12.1 kU_A_/L (IQR, 2.9-35.2 kU_A_/L; range, 0->100 kU_A_/L). The cohort included 115 (41%) children with a history of prior anaphylaxis to egg, of whom 68 (24%) had experienced respiratory symptoms, cardiovascular symptoms, or both with egg ingestion. Seventy-six (27%) were currently tolerating baked egg (eg, in cakes) at the time of enrollment.

### Physician-diagnosed asthma/recurrent wheeze

One hundred eighty-eight (67%) children had a physician's diagnosis of asthma or recurrent wheeze, of whom 145 (51% of total cohort) were using daily preventer therapy (BTS/SIGN step 2 or greater). Sixty-nine (25%) were using high-dose inhaled corticosteroids, multiple preventer therapy, or both. One hundred fifty-seven (56%) had allergic rhinitis, 180 (64%) had atopic eczema, and 138 (49%) were allergic to 3 or more food groups.

### Immediate adverse events after immunization

There were no systemic reactions in the cohort of 282 children. On the basis of these data, the 95% upper CI for the incidence of a systemic allergic reaction (including anaphylaxis) to LAIV in children with egg allergy was 1.3%. The median baseline total ocular and nasal symptom score was 0 (IQR, 0-1); this did not increase at 10, 30, or 60 minutes after LAIV administration (*P* > .05, Wilcoxon signed-rank test).

A total of 14 adverse events in 14 different children were reported within 2 hours of vaccine administration (3.2% of all doses given), 8 of which were consistent with a potential IgE-mediated allergic response, as defined by international consensus.^19^ Thus 2.8% of participants experienced an immediate adverse event after immunization of possible allergic cause. These reactions (6 episodes of rhinitis, 1 episode of localized urticaria, and 1 episode of mild gastrointestinal discomfort) were mild and self-limiting and occurred within 30 minutes of LAIV administration. The remaining events were as follows: 1 episode of fever; 1 child who had a mild flare in eczema 45 minutes after LAIV administration; 2 episodes of nasal obstruction alone without concurrent symptoms of nasal itch/sneezing; and 2 children who had transient, localized, nonspecific skin symptoms (itchy chin without skin signs; 3 nonitchy papules above the upper lip) in the absence of any features to suggest an allergic reaction. All but 1 of these events occurred with the first dose of LAIV. Three of these children received a second dose of LAIV 4 weeks later without reaction.

No risk factors were identified for occurrence of an acute adverse event, allergic or otherwise, when assessed for age, severity of egg allergy, previous influenza vaccination, tolerance to baked egg, and presence of physician-diagnosed asthma/recurrent wheezing or allergic rhinitis (*P* > .05 for all comparisons, Fisher exact test).

Acoustic rhinometry was performed in 13 children: no significant change in minimal cross-sectional area of the nasal airway (suggestive of nasal congestion) was observed (median change in nasal patency, −5.3%; IQR, −18.7% to 18.6%; *P* = .97, Wilcoxon signed-rank test). None of these children reported nasal symptoms.

### Delayed adverse events after immunization

After excluding events in 7 patients that were deemed unrelated or unlikely to be related to vaccination by the independent data monitoring committee, 73 (of 278) children had delayed events (occurring between 2 and 72 hours after vaccine administration) reported after the first dose, and 35 (of 148) had delayed events after the second dose. Across both doses, 91 children had a delayed event after at least 1 dose of LAIV. The delayed events are summarized in Table I. Twenty-six (9.4%; 95% CI for population, 6.2% to 13.4%) children experienced lower respiratory tract symptoms within 72 hours, including 13 (4.7%; 95% CI for population, 2,5% to 7.9%) children with parent-reported wheeze. Children with a diagnosis of recurrent wheeze or asthma were not more likely to experience any adverse events than those without (59/186 [32%] vs 32/92 [35%], respectively; *P* = .68) or wheeze/cough (18/186 [10%] vs 8/92 [9%], *P* = 1.00) after LAIV administration. Wheeze/cough was not more common in children receiving regular inhaled corticosteroid (BTS/SIGN step 2 therapy or greater, *P* = .55). Medical review by the child's primary care physician was sought in 2 cases, but no change in medication or treatment resulted. No child presented to the hospital because of a reported adverse event within 72 hours. No serious adverse events reported during the study were attributable to LAIV administration.

In the 148 children who received 2 doses of LAIV and in whom follow-up was complete, 20 (13.5%) were reported to have experienced an adverse event after immunization within 72 hours for both doses. In only 4 cases were symptoms similar in both reactions, and in 2 of 4 cases the reported adverse event after immunization was an eczema flare.

## Discussion

In this highly atopic cohort of children with egg allergy, there were no systemic allergic reactions or episodes of anaphylaxis after administration of LAIV. This equates to an upper 95% CI of 1.3% for the incidence of an acute systemic allergic reaction for children with egg allergy in the population. Des Roches et al^25^ recently reported a cohort of 68 children with a diagnosis of egg allergy who received LAIV without an allergic reaction; however, the criteria used to define egg allergy in their cohort were less stringent than in the current study, and thus a proportion of the children reported in that study might have no longer been clinically allergic to egg.

The rate observed for attributable allergic reactions after vaccine (2.8%) is higher than previously reported.^10^ These reactions were all mild, localized, and self-limiting. Safety data from postmarketing surveillance in the United States has shown LAIV to be a well-tolerated vaccine.^12-14,26^ Baxter et al^14^ reported 9 episodes of urticaria occurring within 3 days of LAIV administration in children aged 5 to 17 years of 43,702 doses administered during the period 2003-2008. However, it is unclear how many of these episodes occurred within 2 hours of LAIV administration, which is consistent with an IgE-mediated mechanism caused by LAIV. In a further surveillance study of 2.5 million doses of LAIV in adults, 7 cases of systemic allergic reactions (anaphylaxis) occurred, which is equivalent to a rate of 0.3 reactions per 100,000 doses; none were related to egg allergy, and only 5 were deemed to be causally related to LAIV administration.^27^

A randomized, double-blind, placebo-controlled trial of the safety of LAIV in children aged 6 to 59 months without egg allergy reported that the most common adverse event was rhinorrhea/nasal congestion.^10^ This has been subsequently confirmed in postmarketing surveillance studies.^27^ Despite the high rate of atopy in our cohort, the rates of adverse events were similar to those previously reported (Table II).^10^ In this study children with asthma (including those using preventer therapy) or recurrent wheeze were not at greater risk of parent-reported wheeze in the 72 hours after LAIV administration. It was not possible to compare rates of wheeze with those of previous studies because the latter refer to “medically significant wheeze” diagnosed by a health care professional occurring up to 42 days after vaccine administration; unfortunately, rates of parent-reported lower respiratory tract symptoms in the days after LAIV have not been assessed in prior studies. Furthermore, children requiring higher levels of asthma treatment (BTS step 3 or greater) were not at higher risk of parent-reported wheeze, a group that still constituted 25% of our cohort.

A report of 2 randomized multinational trials comparing LAIV with IIV in 1940 children aged 2 to 5 years with asthma or a history of wheezing found no difference between the incidence of wheezing after vaccination between those who received LAIV and IIV.^8^ The rates of lower respiratory tract symptoms ranged from 5% to 29.9% (any wheeze within 42 days of vaccine administration) and are similar to our study. Other studies in older children have likewise found no evidence for an increase in asthma exacerbation or medically significant wheeze after LAIV compared with IIV.^9,16^ It is clear that wheeze is a relatively common symptom in this group of children. In contrast to UK guidance, guidelines in the Unites States and Canada currently recommend against the use of LAIV in children with asthma, although this advice was recently been revised, permitting the use of LAIV in children aged 2 to 4 years without symptoms of wheezing in the 12 months before vaccination.^6^ We found no evidence for an increase in “medically significant wheezing” after LAIV in those children with a history of recurrent wheeze or asthma. We are unable to determine whether the episodes of wheezing observed would have occurred in the absence of LAIV immunization.

Analysis of the 4 batches of LAIV used during this study for the Department of Health, England, found the maximum concentration of ovalbumin present to be less than 0.3 ng/mL. However, the egg protein content of influenza vaccine varies between batches, and our data might not be applicable to future stocks of LAIV in which the egg content of the vaccine might be higher.^28,29^ The maximum level of ovalbumin permitted in LAIV under the license granted by the European Medicines Agency is 1.2 mg/mL^30^; this is approximately 10 times lower than the amount of egg protein found to trigger local rhinitis symptoms when administered into the nasal airways of children with egg allergy.^31^ Therefore it is unlikely that LAIV would be expected to trigger symptoms because of an IgE-mediated allergic reaction to egg.

In summary, these data have demonstrated a safety profile in terms of systemic allergic reactions to LAIV (supplied during the 2013-2014 influenza season) in children with egg allergy, including those with a prior history of anaphylaxis, similar to that previously reported for children without egg allergy. Furthermore, the vaccine appears to be well tolerated in children with a diagnosis of asthma or recurrent wheeze.

**Clinical implications**

Influenza vaccination with LAIV was safe in children with egg allergy, including those with a prior history of anaphylaxis, with no systemic allergic manifestations seen.

## Figures and Tables

**Table I:** Delayed adverse events reported by parents

Delayed symptoms experienced after LAIV	No. of doses	No. of children	Rate in cohort	95% CI for population
Denominator (no. of doses/children in study)	426	278		
Upper respiratory				
Upper respiratory (any)	65	59	21.2%	16.6% to 26.5%
Isolated symptoms only, <24-h duration	23	22	7.9%	5.0% to 11.7%
Isolated symptoms only, >24-h duration	9	9	3.2%	1.5% to 6.1%
Nasal symptoms with ocular involvement	6	6	2.2%	0.8% to 4.6%
Lower respiratory				
Lower respiratory (any)	26	26	9.4%	6.2% to 13.4%
Parent-reported wheeze	13	13	4.7%	2.5% to 7.9%
Constitutional				
Any	31	31	11.2%	7.7% to 15.5%
Fever <24 h	20	20	7.2%	4.4% to 10.9%
Fever >24 h	5	3	1.1%	0.2% to 3.1%
Other: lethargy, headache, dizziness, myalgia	8	8	2.9%	1.3% to 5.6%
Dermatological				
Flare in eczema	13	11	4.0%	2.0% to 7.0%
Nonspecific rash, no response to antihistamine	2	2	0.7%	0.1% to 2.6%
Abdominal symptoms				
Vomiting, nausea, abdominal pain	11	11	4.0%	2.0% to 7.0%
Loose stools	6	6	2.2%	0.8% to 4.6%
Ear-nose-throat				
Mild epistaxis	1	1	0.4%	0.01% to 4.6%
Ocular				
Itch, redness	8	8	2.9%	1.3% to 5.6%
Neurological				
Any	0	0	0%	0% to 1.3%
Cardiovascular				
Any	0	0	0%	0% to 1.3%

**Table II:** Rates of adverse events occurring within 72 hours after LAIV administration in this study compared with published rates in the literature

Symptoms within 72 h	Current study		Reported
Allergic reaction (mild symptoms) only	9/433	2.1%	0.02%
Allergic reaction: anaphylaxis	0/433	0%	0%
Fever	25/426	5.9%	5.4%
Nasal symptoms	65/426	15.3%	31%
Wheeze (parent reported)	13/426	3.1%	Not reported
Wheeze requiring treatment by physician	0/426	0%	0.2%
Lower respiratory symptoms	26/426	6.1%	Not reported
Eczema flare	13/426	3.1%	Not reported

Rates are reported as a proportion of the total number of doses given to be consistent with the method of reporting used in the existing literature.^10^
